# Back to basics: reflective take on role of MCQs in undergraduate Malaysian dental professional qualifying exams

**DOI:** 10.3389/fmed.2023.1287924

**Published:** 2023-11-30

**Authors:** Avita Rath

**Affiliations:** ^1^Faculty of Dentistry, SEGi University, Petaling Jaya, Selangor, Malaysia; ^2^Edinburgh Medical School- Clinical Education, University of Edinburgh, Edinburgh, United Kingdom

**Keywords:** dental education, assessment, MCQ assessment, high-stake tests, undergraduate

## Introduction

The Bachelor's Dental Programme (BDS) in Malaysia is a 5-year full-time undergraduate course, the tenets of which lie in an overarching competency- and outcome-based curriculum (1). It aims to prepare dental students to become independent, reflective practitioners who deliver quality patient care (2). The programme aims at organizing the graduates' attributes around a wide range of competencies that include evidence-based knowledge, critical thinking, problem-solving, procedural skills, ethical values, and professionalism (3, 4). It also emphasizes student-centered learning and provides a design-down framework based on attainable learning objectives that drive the pedagogy/instructions reflected in an authentic assessment (5, 6).

The final summative assessment, or the professional examination, is usually a combination of written and performance-based formats that aim to measure the different facets of competencies in alignment with the course goals as per the Malaysian Dental Council guidelines (3). Written assessments that entail 60% of the final grades have long prevailed in assessments to capture the cognitive domains of Bloom's Taxonomy that may span from knowledge recall to evaluation or capture the “knows and knows-how” of Miller's pyramid of competency (4, 7, 8). Among other formats, multiple choice questions (MCQ) is the most sought-after design for these forms of assessments. Albeit known for their ubiquitous presence due to their testing breadth of knowledge and ease of administration, they are spuriously known to defy recommended guidelines and have garnered a negative reputation for engaging lower cognitive domains or even the test-wiseness ability in lieu of actual knowledge (9, 10). Our existing MCQ paper consists of 60 items of one-correct answer (OCA) with four options and complex two-tier or K-type questions that predominantly assess rote recall. Supposedly, if the final summative assessment provides legitimacy by certifying the measured competencies (11), in that case, the predictive accuracy of an assessment toward measured competency (12) may be questionable, putting the quality of the entire programme at risk and prompting immediate action (13). Moreover, under the new dental act (14), the graduates must appear for a professional qualifying examination (PQE), a licensing exam with single best answers (SBA), and an objective structured clinical examination (OSCE) format commencing in 2025, to register for practice (14). Hence, it seems incumbent to go back to basics, revisit MCQ for its worth as an authentic assessment tool, and take a pragmatic approach contingent on its pros and cons, its acquiescence with other assessment formats, and its fitness for the purpose of qualifying exams for courses like dentistry such as ours.

## Purposes of the assessment

Boud famously stipulated that “assessment always does double duty” (15). Based on the stakes involved, those duties/purposes could be broadly divided as formative or assessment for learning, which are low-stakes, ongoing, address the gaps, and assimilate notions construed by the learners by re-clarifying the learning outcomes. Whereas summative or assessment of learning is applied at the end of a module or the course itself. It forms the crux for high-stakes decisions to pass or fail. The data accrued from these assessments further typifies the programme evaluation and holds accountability to stakeholders (16).

In reality, there is always “a continuum of summative to formative…, depending on the primary intended purpose” (17). Therefore, the goals of an assessment tool are contingent on its purpose, which influences its content and strategies (18).

Any assessment tool is informed by a fair share of strengths and weaknesses (19). Hence its utility (U) or usefulness, a conceptual layout posited by van der Vleuten, is a function of the prescribed criteria of reliability (R), validity (V), cost (C), acceptability (A), and educational impact (E), wherein the *weighting* (w) of each component is akin to the purpose of the assessment (20).


$$\begin{array}{l} U_{W}=R_{W}\times V_{W}\;\times \;C_{W}\times A_{W}\times E_{W}. \end{array}$$

Thus, from the vantage point of this enduring framework and other literature is the inductivist way to critically appraise the purpose of the revised tool (21).

An indispensable criterion of high-stakes assessment is the reliability or reproducibility of the scores (20), which is also associated with the validity of its internal structure (17). Often expressed as Cronbach's alpha coefficient (α; ranging from 0 to 1), the values above 0.8 are deemed acceptable for high-stakes exams (22).

Evidence suggests that MCQ formats are renowned for their high reliability (23–25). A common misconception was that its high reliability was due to its objectivity (26). On the contrary, high reliability is borne out of an adequate sampling of questions and as a function of testing time (20).

A possible suggestion of well-designed SBA items over 2–3 h in place of the current 1.75 h 60-item K-type MCQ paper may demonstrate high reliability, as shown in my recent study in the medical education context (27, 28), and must be considered for future assessments.

Conversely, I would like to highlight the hazards of confining reliability to the numeric α alone. It only expresses the degree of replicability of the rank order of candidates or the internal consistency of the scores (29) and doesn't recognize the discriminating power in performances, a primacy for high-stake decisions (17). It is the discrimination index (DI) that describes the discernment capability of an item to differentiate between scorers based on their proficiency in the tested domain (30). Ranging from −1 to +1, which traditionally corresponds to the top and bottom 27% of the cohort, a DI of ≥0.3 for 50–60 items probably would give good reliability (17). The main enemies of DI are the item writing flaws (IWF), such as the implausible or non-functioning distractors (NFDs), *one* of the common rogues in the existing tool (31). NFDs are an option(s) of a question other than the correct answer, which is generally selected by < 5% of the examinees (A-value) and illustrated as trace lines (26).

The handed-down format of previous years with 4- or 5-options has never been investigated for IWFs, indicating a heuristic mentality that more options imply increased difficulty with reduced guessing or cueing effect, which may be true provided there were no IWF (32). Moreover, an 80-year meta-analysis clarifies that it is not feasible for more than three plausible distractors and that it would suffice the DI of an MCQ paper (33). Nevertheless, research has shown that a variable number of options based on their educational availability would bolster the content validity of the item, concurrently strengthening its reliability and underpinning my recommendation (34, 35).

Furthermore, DI is also related to an item's difficulty or facility index (*P*-value), expressed in the range of 0 to 1.0, where a higher *P*-value denotes an easier question. Data entail that the SBA tool should have a moderate range of *P*-values (0.25–0.75) to foster a good DI (30). Having said that, some of the items may be defined by learning outcomes that assess the lower levels of cognition and are ostensibly easy for final-year students. Conversely, too many of these items, as seen in the existing tool, are predisposed to higher IWFs and would deter the high performers, threatening the tool's validity (10, 30, 36).

Alongside reliability, validity is another fundamental attribute of the summative exam, which concerns whether the scores measure the competency it purports to (12, 17). A caveat to note is that reliability is a prerequisite to the validity of an assessment; however, it does not ascertain its validity (37).

Modern concepts of validity are overarching and posit a “unitary” framework based on the premise of the fidelity of scores and their inferences (38, 39). I will be highlighting the pertinent concepts with a few mentions of others within the constraints of the space here. Foremost is based on the content of the assessment tool, which should be constructively aligned with the learning outcomes of the topics (29, 40). This is ensured through blueprinting, a method where the test items are mapped against the relevant learning objectives set at appropriate taxonomic levels prior to the commencement of the academic year (18). It apprehends the threat of construct under-representation (CUR)—under-sampling or oversampling of the course content (41). In spite of an entrenched blueprint in our faculty, CUR issues have been noticed, especially in a theme-based MCQ paper when there is an overcompensation by items from feasible topics or when the existing items that are nominated for higher cognitive levels tend to elicit factual recalls (41). Consequently, I would want to paraphrase Coderre's opinions here, which state that audit adherence to the blueprint is required and that creating it alone is insufficient (42). Every item of the new tool should be evaluated for its accurate representation and suitability of the learning outcome for fairer and more reliable scores (43).

Validity is a nebulous term, especially for the critics of the MCQs, who question the authenticity of this close-ended design in eliciting clinical reasoning, which is more nuanced than just selecting an option (44, 45). I acknowledge the connotations of these arguments; nevertheless, one should bear in mind that the inability to record the reasoning processes does not insinuate their absence (46). Moreover, authenticity is present at all levels of the pyramid (37). Based on this conclusion, as clinical reasoning requires integrative knowledge that entails high-order cognitive skills of application and analysis (18), the new tool with well-designed clinical vignettes could invoke this domain of human endeavor regardless of the response format (47).

That said, the veracity of the stimulus generated could be eluded by errors or “noise” in items leading to the construction of irrelevant variance (CIV) such as grammatical chicanery, complex language, and pseudo-vignettes in a trivial pursuit of elusive “blueprint alignment” draining its fitness in summative exams (17, 48). With no intentions to gainsay written formats that are susceptible to CIV (23, 49), the feasibility of obviating a CIV in an MCQ is higher owing to its compact design (36, 50).

Facets of validity also converge with other utility criteria and would permeate the next section of this discussion. For instance, the *consequential validity* is somewhat analogous to the educational impact (17). A deep-set reality initially underscored by van der Vleuten was that “*Assessment drives learning*” through its content, format, timing, and feedback (20), especially when the summative culture looms large. One must understand that students are *agentic* learners who always prioritize their learning around exams. My take is to be astute and capitalize on these drivers by focusing on the design choices of the new SBA and its strategic placement within the toolkit that would determine its influences within the precincts of the programmatic assessment (21).

## Design choices

It seems axiomatic that the educational impact of an assessment is inextricably linked to the assessment literacy of stakeholders, which might be scarce in my setting. Every student at our faculty (SEGi University) owns a handbook with the layout of the assessment structure. However, there is a lack of emphasis on a meta-dialogue early on about the purposes and function (51), as most faculty are at the outset of the curriculum and assessment (52). Although we have had a few cursory workshops, marshaling nuggets of information, the insights are tentative and might have negative implications for the fairness of this tool. In my view, fairness is more of an annotation of the assessment process itself than a design choice, so it is quixotic to address it. To a great extent, it is associated with the stakeholders' acceptability and other utility criteria (24).

Keeping the good name of the new SBA format requires early intervention at the item development stage to avoid CIVs and CURs, as seen in the previous section. As item writing has always been referred to as an art (53), to improvise and excel, it calls for extensive training for most of us who have an intuitive idea of suboptimal design but lack the acumen to identify it. But that would incur a cost—not just economic, but faculty's time—enshrined beliefs further restrained by university policies. Even so, weighing the cost relative to its purpose (20) asserts training to be a worthy investment in the long haul of superior assessments.

Research also asperses the format for inducing adverse *testing effects* where an incorrect answer choice lures the examinees to recall their facts wrong for other exams (45). Albeit not wholly avoidable owing to the selected response design, the scheduling of the paper might mitigate this issue to some extent. Currently, the MCQ is the last paper, which seems suitable for the revised tool, as the deep learning expended around other formats should generate a positive testing effect (54). However, one can never predict the educational impact of assessments without thorough screening and follow-up (20). In fact, to assess the fitness of the new tool, there need to be qualitative pre-assessment and post-assessment checks.

Albeit a *de-facto* review process that occurs at the subject, faculty, and external examiners' stages, it might fortify the quality assurance of the questions at a speciality and interdisciplinary level (55, 56). There is a possibility to make these sessions more defensible and credible so that assessment practices are more legitimate and a good fit for the cohort and the curriculum by mandating standard-setting and item analyses (57).

Standard is a conceptual boundary on a “true” cut-score scale that differentiates between acceptable and non-acceptable performance; in other words, optimal or passing standards can be viewed as an agreed definition of competence that reflects expert judgement as to what constitutes it, backed by several sources of evidence (58). Based on Kane's view of valid inferences (59), relevancy evaluations of an assessment to a well-mapped blueprint are prerequisites to setting standards. It delineates what a competent student needs to know vs. what they must know about a construct, as cited in Schuwirth and van der Vleuten (60). This allows for setting a cut-off or passing score on an observed score scale that should be used to make a defensible, deliberate judgement for that relevant competence. For an SBA tool, a criterion-referenced or absolute test-centered standard setting such as the modified Angoff method is the most appealing as the judgements are made on individual items based on item analyses in the backdrop of minimal competence. It also gives wiggle room for discussion and consensus around the performance data (61, 62).

Nevertheless, practice exercises in the course revealed that it is not possible without psychometric experts, who could employ the correct model for it. Classical test theory (CTT), which consists of α, *P*-value, DI, and *A*-value, discussed earlier, sits well here due to its uni-dimensional construct and simple statistical software (63). Moreover, judgements can be fallible and time-consuming due to a lack of expertise, so it requires the selection of a judges' panel of every age and gender with knowledge of the curriculum. Those who could articulate characteristics of a “minimally competent” based on the cohort's abilities were at the “borderline” of pass and fail (64, 65).

We currently follow an absolute standard of 50% pass-score based on a compensatory method that combines all the formats to produce average marks translated to grades. Evidence reveals that combining scores across the papers to moderate the errors of individual formats is highly reliable for high-stakes decisions (26). Conversely, this method may induce a minimalist study strategy, wherein past students have passed by doing well in specific papers alone. But this point of view imparts a reductionist approach toward competency. Pioneers drew on these issues and espoused a programmatic approach toward assessment (37) that pleads on the holistic narrative of competency, vying that “any single assessment is a weak data point and implies a compromise on the quality criteria” (20). It is always recommended to deploy a deliberate suite of assessments that ameliorates the trade-offs of the utility of various formats, such that collated information is more than the sum of its parts (19). This principle underlies the assumption of triangulating data from multiple sources and formats throughout the year based on domain specificity, providing robust, meaningful conclusions toward competency rather than relying on a single format (21).

Since the exam is a recursive process, it is also important to perform post-exam item analyses, which would corroborate the credibility and defensibility of the assessment (66). The exercise would yield meaningful feedback for future pre-assessment analyses, identify errors in unfair scores, and, most importantly, justify the need for remediation through the resit exam. Usually, our faculty allows a single resit opportunity after an exit exam within 2 weeks of the final results; however, the ideal number of attempts is debatable (67). Considering the limited faculty resources, especially with a PQE lurking in a few weeks, a single resit looks like the only option for now. Moreover, the advent of PQE seems promising toward desirable but nearly absent catalytic effect or educational feedback (68) from an exit exam (69).

To paraphrase, the possibility of a “fairy-tale” assessment is the wrong question to start with. The burgeoning assessment literature has revealed that there is no ideal tool as they are not goals in themselves, not even my proposed tool. Nonetheless, despite the format's long pedigree, its (over)usage should be monitored in the context of the programme. In summary, the gargantuan responsibility of assessment tools to credibly answer the relentless inquiry of “how much is good enough?” is an outdated pursuit. Thus, it is no longer a question of measurement but an integral issue of the curricular design and the users' expertise in the organizational culture. Moreover, I would contend that the system of continuous longitudinal assessment must be designed with an attempt to operationalize a programmatic assessment format broadly aligned with principles suggested by the proponents of assessment philosophy as discussed above if we wish that assessment to provide authentic information about our learners and their progress milestones in the continuum of their professional development as budding dental professionals.

## Author contributions

AR: Conceptualization, Project administration, Resources, Writing—original draft, Writing—review & editing.
